# Satiety Factors Oleoylethanolamide, Stearoylethanolamide, and Palmitoylethanolamide in Mother’s Milk Are Strongly Associated with Infant Weight at Four Months of Age—Data from the Odense Child Cohort

**DOI:** 10.3390/nu10111747

**Published:** 2018-11-13

**Authors:** Signe Bruun, Sandra Gouveia-Figueira, Magnus Domellöf, Steffen Husby, Lotte Neergaard Jacobsen, Kim F. Michaelsen, Christopher J. Fowler, Gitte Zachariassen

**Affiliations:** 1 Strategic Business Unit Pediatric, Arla Foods Ingredients Group P/S, DK-8260 Viby J, Denmark; lotte.neergaard.jacobsen@arlafoods.com; 2 Hans Christian Andersen Children’s Hospital, Odense University Hospital, DK-5000 Odense C, Denmark; steffen.husby@rsyd.dk (S.H.); gitte.zachariassen@rsyd.dk (G.Z.); 3 Department of Clinical Research, Faculty of Health Sciences, University of Southern Denmark, DK-5000 Odense C, Denmark; 4 Clinical Chemistry, County Council of Västerbotten, SE-901 85 Umeå, Sweden; sandra.gouveia@vll.se; 5 Department of Clinical Sciences, Pediatrics, Umeå University, SE-901 87 Umeå, Sweden; magnus.domellof@umu.se; 6 Department of Nutrition, Exercise and Sports, Faculty of Science, University of Copenhagen, DK-1958 Frederiksberg C, Denmark; kfm@nexs.ku.dk; 7 Department of Pharmacology and Clinical Neuroscience, Umeå University, SE-901 87 Umeå, Sweden; christopher.fowler@umu.se

**Keywords:** infant growth, appetite regulation, *N*-acylethanolamines, OEA, SEA, PEA, breastfeeding, human milk composition, obesity

## Abstract

Regulation of appetite and food intake is partly regulated by *N*-acylethanolamine lipids oleoylethanolamide (OEA), stearoylethanolamide (SEA), and palmitoylethanolamide (PEA), which induce satiety through endogenous formation in the small intestine upon feeding, but also when orally or systemic administered. OEA, SEA, and PEA are present in human milk, and we hypothesized that the content of OEA, SEA, and PEA in mother’s milk differed for infants being heavy (high weight-for-age Z-score (WAZ)) or light (low WAZ) at time of milk sample collection. Ultra-high performance liquid chromatography-mass spectrometry was used to determine the concentration of OEA, SEA, and PEA in milk samples collected four months postpartum from mothers to high (*n* = 50) or low (*n* = 50) WAZ infants. Associations between OEA, SEA, and PEA concentration and infant anthropometry at four months of age as well as growth from birth were investigated using linear and logistic regression analyses, adjusted for birth weight, early infant formula supplementation, and maternal pre-pregnancy body mass index. Mean OEA, SEA, and PEA concentrations were lower in the high compared to the low WAZ group (all *p* < 0.02), and a higher concentration of SEA was associated with lower anthropometric measures, e.g., triceps skinfold thickness (mm) (β = −2.235, 95% CI = −4.04, −0.43, *p* = 0.016), and weight gain per day since birth (g) (β = −8.169, 95% CI = −15.26, −1.08, *p* = 0.024). This raises the possibility, that the content of satiety factors OEA, SEA, and PEA in human milk may affect infant growth.

## 1. Introduction

Globally, the prevalence of obesity has tripled since 1975 including a dramatic increase in the prevalence of overweight and obesity in children. In 2016, 41 million children under the age of 5 years, and more than 340 million children and adolescents aged 5–19 years were overweight or obese [1]. It is pronounced and acknowledged that rapid or excess weight gain, most often defined as a change in weight-for-age Z-score (WAZ) of ≥0.67 [2,3], during the first two years of life is associated with a higher risk of being overweight or obese in later childhood [4], and that the association is even more pronounced for rapid weight gain during the first year of life [5]. Furthermore, childhood overweight and obesity track into adolescence and adulthood [6], resulting in an increased risk of noncommunicable diseases (e.g., cardiovascular diseases, diabetes, cancer) and premature death [1]. Increased intake of energy-dense foods (including a transition to a higher n-6/n-3 ratio of the dietary polyunsaturated fatty acids intake [7]) and decreased levels of physical activity are major elements in the ongoing obesity epidemic.

In parallel, research and knowledge on appetite regulation has increased in the past decade. Appetite regulation is multifactorial, but—extremely simplified—appetite and energy homeostasis are regulated via stimulatory (orexigenic) or inhibitory (anorexigenic) signaling pathways, which are active in the central nervous system (CNS) in concert with the gastrointestinal system, adipose tissue (leptin, adiponectin), and the pancreas [8,9].

The regulation of appetite and food intake is partly regulated by the *N*-acylethanolamine (NAE) lipid oleoylethanolamide (OEA) [10]. OEA acts peripherally and causes a state of satiety accompanied by prolonged inter-meal intervals, reduced size of feedings, and increased fatty acid uptake via interaction with the peroxisome proliferator-activated receptor α (PPAR-α) and the transient receptor potential cation channel vanilloid-1 (TRPV1), which stimulate the vagal nerve [11], thereby indirectly signaling satiety to the hypothalamic nuclei [12]. However, the involvement of the vagal nerve is debated [13], and delayed gastric emptying and intestinal transit are other mechanisms described; independent of both PPAR-α and TRPV1 [14].

It is well described that feeding (especially dietary fat) promotes or activates the endogenous formation of OEA in the enterocytes of the small intestine, but (at least in rodents) orally or systemic administered OEA has shown effects similar to those of endogenous OEA, i.e., inducing satiety [14]. Related NAE lipids such as stearoylethanolamide (SEA) and the anti-inflammatory palmitoylethanolamide (PEA) have shown appetite-reducing effects in animal models, though these findings are less consistent and the effects may be weaker than those of OEA [10,15,16,17].

Human milk is considered ideal and appropriate as the only food for the first six months of life [18], since it contains a variety of components essential for infant growth, development and well-being, e.g., vitamins, minerals, carbohydrates, amino acids and proteins, hormones, growth factors, and antimicrobial factors. In the late 1990s, OEA, SEA, PEA, and other NAE lipids were detected in both human and animal milk [19,20,21].

The fact that orally administered OEA (and to some extent SEA and PEA) exert some of the same effects as endogenous OEA raises the possibility that the presence of these lipids in human milk play a role in the regulation of appetite and food intake in breastfed infants. In the present study, we aimed to determine the concentration of OEA, SEA, and PEA in human milk samples collected four months postpartum and investigate possible associations between the levels and concurrent infant anthropometry and growth from birth. Our hypothesis was that the concentration of the NAE lipids OEA, SEA, and PEA in mother’s milk would differ between infants being relatively heavy at the time of the milk sample collection (i.e., having a high WAZ) and infants being relatively light (i.e., having a low WAZ).

## 2. Materials and Methods

### 2.1. Participants and Milk Sample Collection

The Odense Child Cohort is an unselected, prospective birth cohort comprising infants born in the municipality of Odense, Denmark [22]. From January 2010 to December 2012, pregnant women of gestation <16 weeks were invited to participate; the only exclusion criterion was emigration from the municipality of Odense before birth. From March 2012, inclusion was extended to 2.5 months postpartum, but the majority of participants were included during pregnancy. The study consists of self-administered questionnaires and physical examinations including collection of biological material (blood samples, fecal samples, etc.) at 4 and 18 months, 3, 5, and 7 years of age. Further questionnaires and examinations are planned (yet not initiated) at 9, 12, 15, and 18 years of age.

As part of the physical examination, anthropometric data are collected in terms of e.g., length, weight, triceps and subscapular skinfold thickness. Abdominal circumference was measured in cm (one decimal) using a measuring tape (seca 212, Seca, Hamburg, Germany). Weight was measured in g (no decimals) using an electronic baby scale (seca 717, Germany). Length was measured in cm (one decimal) using a baby measuring rod (seca 231, Germany). Triceps and subscapular skinfold thickness was measured in mm (one decimal) using a skinfold caliper (Harpenden Skinfold Caliper, Baty International, West Sussex, England); the measurement was repeated three times, and the arithmetic mean calculated. In addition, 13 children in 2012 and 7 children in 2013 were measured by two examiners to determine inter-observer agreement. WAZ, height-for-age Z-score (HAZ), BMI-for-age Z-score (BMIZ), and weight-for-height Z-score (WHZ) are calculated based on the WHO’s 2006 standards using the official STATA module, zscore06 [23].

From April 2012 and onwards, mothers were given the opportunity to deliver a milk sample, when their infant was seen for the first physical examination at four months of age. The mothers were to express the sample in the clinic, and a total of 30 mL was requested, but less was accepted. All physical examinations and thereby sample deliveries were scheduled between 8 a.m. and 5 p.m. There were no requirements regarding the sample being fore or hind milk; the use of a breast pump or manual expression; or recording of time since last feeding.

**Figure 1:**
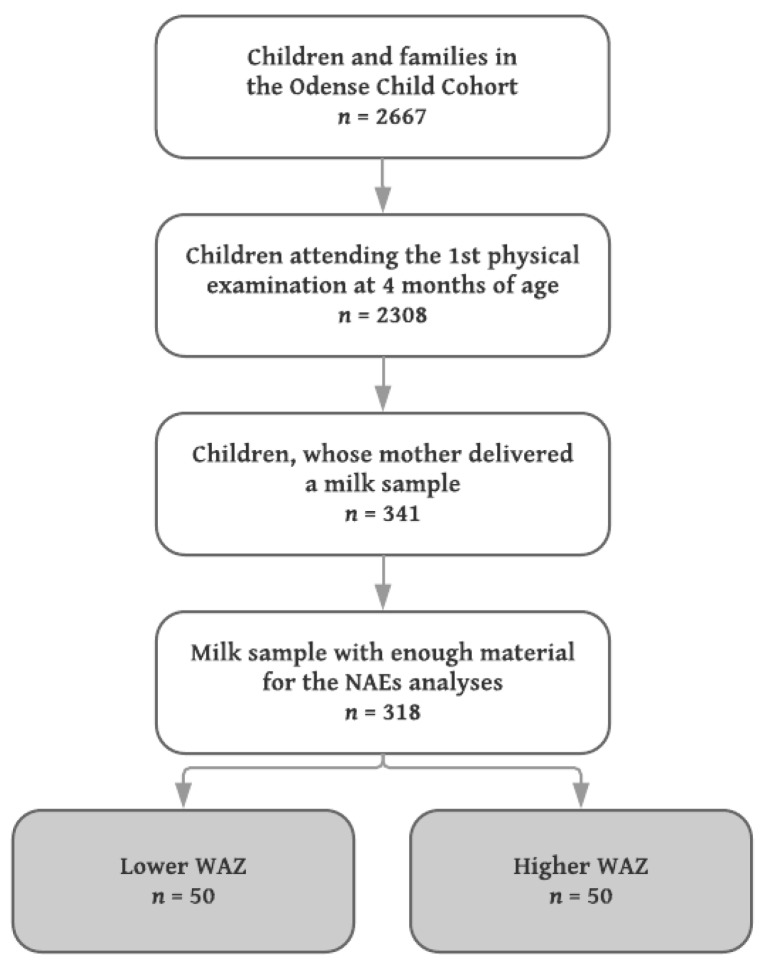
Flowchart of inclusion in the present study. WAZ: weight-for-age Z-score.

Milk samples from mothers to singleton infants with the lowest (*n* = 50) and highest (*n* = 50) WAZ were included, median (IQR) WAZ −0.67 (−0.98, −0.54) and 1.52 (1.31, 1.84), respectively. Since no a priori data concerning differences in the levels of NAE lipids in human milk exist, a power calculation was not performed, but the sample size chosen was considered sufficient to detect robust changes of biological (as opposed to purely statistical) significance. A flowchart of the inclusion is shown in Figure 1.

Directly upon milk sample collection, the sample was split into 10 mL tubes (100 × 16PP, Sarstedt, Nümbrecht, Germany) and stored at 5 °C. Within a maximum of three days after delivery and if at least 10 mL had been delivered, macronutrient analysis regarding the content of protein, fat, lactose, and energy (g/100 mL and kJ/100 mL, respectively) was performed (Miris HMA, Uppsala, Sweden); otherwise, macronutrient analysis was not prioritized. The remaining sample was centrifuged at 3600 rpm and 21 °C for 5 min. (Eppendorf Centrifuge 5702 R, Eppendorf Corporate, Wesseling-Berzdorf, Germany). The resulting fat, skimmed, and solid fractions were manually aliquoted (3.5 mL transfer pipette, Sarstedt) into three different tubes (3.6 mL Nunc^®^ CryoTubes^®^, Thermo Fisher Scientific, Waltham, MA, USA) and stored at −80 °C. The skimmed fractions were shipped from Odense, Denmark to Umeå, Sweden on dry ice and remained frozen (−80 °C) upon arrival.

### 2.2. Analysis and Quantification of NAE Lipids

Analysis was performed according to a previously validated and published method [24]. The samples had to be centrifuged prior to solid-phase extraction (SPE), since some particles were left in the suspension. In brief, the samples (spiked with 20 µL internal standard (IS) solution at a concentration of 20 ng/mL for OEA-d4, SEA-d3, and PEA-d4) were applied to the SPE columns, and then washed by a solution of 5% methanol with 0.1% acetic acid. Afterwards, the metabolites were eluted using 2 mL of acetonitrile and 2 mL of methanol. Finally, the samples were dried using speed vacuum; reconstituted in 100 µL of methanol, and spiked with 10 µL of the recovery standard 12-[[(cyclohexylamino)carbonyl]amino]-dodecanoic acid (CUDA; 0.025 µg/mL). An additional centrifugation was done with an Eppendorf tube filter.

The quantification was carried out by means of ultra-high performance liquid chromatography-mass spectrometry (UPLC-MS). The system used was Agilent UPLC system (Infinity 1290) coupled with an electrospray ionization source (ESI) to an Agilent 6490 Triple Quadrupole system equipped with the iFunnel Technology (Agilent Technologies, Santa Clara, CA, USA), operating in positive mode. Metabolites separation was performed using a Waters BEH C18 column (2.1 × 150 mm, 130Å, 1.7 μm particle size). A flow rate of 300 μL/min. and 10 μL injection volume were employed. The mobile phase consisted of (A) 0.1% acetic acid in MilliQ water and (B) acetonitrile:isopropanol (90:10), and the following gradient was used: 0.0–2.0 min, 30–45% B; 2.0–2.5 min, 45–79% B; 2.5–11.5 min, 79% B; 11.5–12 min, 79–90% B; 12–14 min, 90% B; 14–14.5 min, 90–79% B; 14.5–15.5 min, 79% B; 15.6–19 min, 30% B. ESI applied conditions were optimized as described elsewhere [24]. The MassHunter Workstation software was used to control the instrument and to integrate all peaks manually.

The quantification was achieved by preparing a 10-point calibration curve using pure quantification standards. Furthermore, the recovery rates of each IS were calculated by adding the recovery standard (CUDA) to each sample. Using this technique, we were able robustly to quantify OEA, SEA, and PEA as well as other lower-abundance NAEs including the endocannabinoid anandamide (AEA). The related endocannabinoid 2-arachidonoylglycerol (2-AG), which belongs to the monoacylglycerol class of lipids, was also robustly measured. We decided not to present data on AEA and 2-AG due to their high degree of sensitivity to sample storage conditions including a rapid ex vivo synthesis and/or release from cells present in the samples prior to freezing [20,25,26]. The recovery rates (in %) of each NAE were investigated using the IS, presented as mean ± SD for low vs. high WAZ group respectively, *p* values are for Welch’s two-sample *t*-test: OEA, 44 ± 20 vs. 51 ± 18, *p* = 0.070; SEA, 28 ± 14 vs. 33 ± 13, *p* = 0.073; PEA, 33 ± 16 vs. 40 ± 15, *p* = 0.038. As a consequence, the individual values reported in the following were corrected for recovery to negate this variability.

The following standards and internal standards were purchased from Cayman Chemicals (Ann Arbor, MI, USA); OEA, SEA, PEA, OEA-d4, SEA-d3, PEA-d4, and CUDA. Acetonitrile and methanol were purchased from Merck (Darmstadt, Germany), isopropanol from VWR PROLABO (Fontenay-sous-Bois, France), and acetic acid from Aldrich Chemical Company, Inc. (Milwaukee, WI, USA). All solvents and chemicals were of HPLC grade or higher. Water was purified by a Milli-Q Gradient system from Millipore (Milford, MA, USA), now Merck (Darmstadt, Germany). Oasis HLB cartridges (60 mg) were obtained from Waters (Milford, MA, USA).

### 2.3. Ethics

The study was approved by The Danish Data Protection Agency (ref. 12/26892), The Regional Committees on Health Research Ethics for Southern Denmark (ref. S-20090130, sub protocols 12, 18, and 37), and complied with the World Medical Association’s Declaration of Helsinki II.

### 2.4. Statistics

Descriptive statistics were performed to describe the participating mother-infant dyads, categorized as either low or high WAZ. Continuous variables included were maternal pre-pregnancy BMI (termed mBMI, kg/m^2^), gestational age (days), birth weight (g), infant weight at the time of milk sample collection (g), infant age at milk sample collection (weeks), and duration of exclusive breastfeeding (weeks), the latter based on weekly text message questions as recently described elsewhere [26]. Dichotomous or categorical variables were maternal educational level (three categories; low, intermediate, and high), maternal smoking (yes or no, the latter including those who stopped smoking during the first trimester), birth type (vaginal birth or Caesarean section), postdelivery parity (three categories; 1, 2, or ≥3), sex (male or female), supplementation with infant formula within breastfeeding establishment; i.e., in the first few days after birth, but not necessarily later on (termed early infant formula, yes or no) [27], exclusive breastfeeding at the time of milk sample collection (yes or no) [27], and season at milk sample collection (either October–March or April–September). The latter was included, since we hypothesized that maternal dietary intake [28] and/or use of medications could differ between seasons, thereby influencing the NAE levels [29].

Maternal and infant baseline characteristics were compared using either two-sided *t*-test for normal distributed continuous variables; two-sample Kolmogorov–Smirnov test for not normal distributed continuous variables (comparing the cumulative distributions of data between the two WAZ groups); or Fisher’s exact test for categorical variables. Normality of continuous variables were tested using Shapiro–Wilk W test.

To investigate the association between milk NAE concentration and WAZ group, a two-way ANOVA was run, examining the effect of WAZ group (low or high), NAE structure (OEA, SEA, or PEA, respectively), and the interaction WAZ group × NAE structure on the NAE levels. The ANOVA matched the NAE structures.

As an alternative approach, receiver operating characteristics (ROC) curves were constructed, and areas under the curve (AUC) were calculated to determine, whether or not the milk NAE concentration could discriminate the two WAZ groups. This approach is non-parametric in nature, therefore not sensitive to the (lack of) normality of the distribution. The AUC can range from 0.5 (no discriminatory power) to 1 (perfect discriminatory power) [30].

Finally, to investigate the correlations between the NAEs of interest and other milk components as well as maternal and infant characteristics, Spearman’s correlation coefficients ρ (rho) were calculated.

The inclusion of covariates in the adjusted analyses was based on the descriptive statistics, and covariates included were birth weight (data from *n* = 100), early infant formula (*n* = 81), and mBMI (*n* = 100).

As the primary inferential statistical analysis, associations between milk NAE concentration and WAZ groups were investigated using adjusted logistic regression analysis. As the secondary analysis, associations between milk NAE level and each of the following outcomes were investigated using adjusted linear regression analyses; abdominal circumference, weight, length, triceps skinfold thickness, subscapular skinfold thickness, WAZ, HAZ, WHZ, BMIZ, total weight gain since birth (Δ weight), weight gain per day (Δ weight per day) since birth, and change from birth weight Z-score to WAZ at four months (ΔWAZ). Lastly, stratification by sex was considered by comparing stratified results with overall results.

Level of significance was set at α < 0.05. However, due to multiple testing and risk of false positives, a false discovery rate of 5% was pre-defined, and the Benjamini and Hochberg procedure was used to calculate the critical value of *p* [31]. In consequence, and where appropriate, we have shown the unadjusted *p* and indicated the critical value of *p* in text, tables, and figures.

Descriptive and inferential statistics were conducted using STATA IC/15.1 (College Station, TX, USA), R Statistical Program vers. 3.4.1 (R Core Team, 2017), and GraphPad Prism 7.0b for Macintosh (GraphPad Software Inc., San Diego, CA, USA).

## 3. Results

### 3.1. Participants

An overview of maternal and infant characteristics across the two WAZ groups is shown in Table 1.

The two groups were well-matched regarding maternal educational level, smoking status, birth type, gestational age, postdelivery parity, infant sex, duration of exclusive breastfeeding, and—at the time of milk sample collection—infant age, season, and breastfeeding exclusivity. However, the two groups differed significantly regarding birth weight, mBMI, and number of infants supplemented with infant formula within breastfeeding establishment—i.e., early infant formula—but regarding the latter two, *p*-values were higher than the critical value of *p* = 0.008, and should be considered in this light.

### 3.2. NAEs

**Figure 2:**
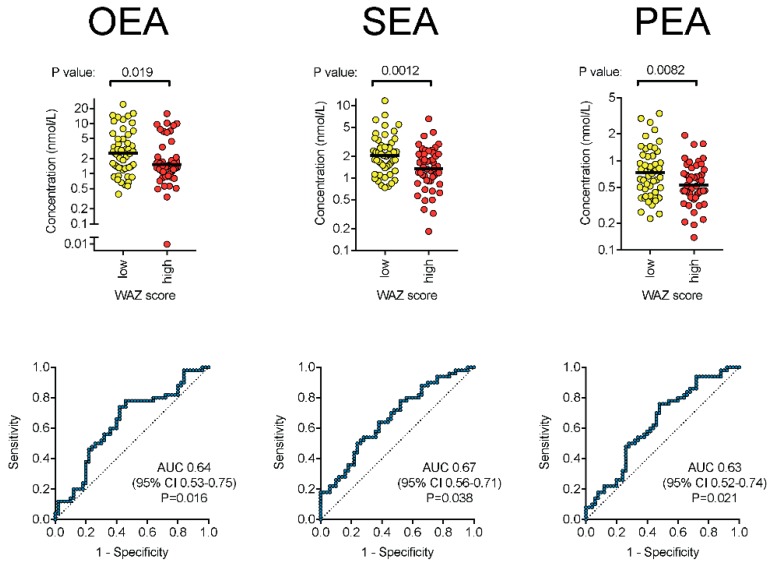
Oleoyl-(OEA), stearoyl-(SEA), and palmitoyl-(PEA) ethanolamine levels in milk samples.

The individual values for OEA, SEA, and PEA are shown in the top row graphs in Figure 2. The upper panels show scatter plots (on a log_10_ scale) for the low and high WAZ groups (both *n* = 50), with the mean of the logged values being indicated by the bars. *p*-values are for Welch’s two-sample *t*-tests. The lower panels show the ROC analyses for each lipid with area under the curve (AUC), 95% CI, and *p*-values being given in each graph. There is a possible outlier for OEA, but the *P*-value remained significant (0.035, Welch’s two-sample *t*-test) upon removal of this outlier. The critical value of *p* at a false discovery rate of 5% was 0.05.

The median (IQR) concentration was 1.54 (1.04–3.97) nmol/L for OEA, 1.7 (1.10–2.44) nmol/L for SEA, and 0.61 (0.41–0.91) nmol/L for PEA. Due to non-normality of residuals, results were log_10_ transformed prior to statistical analysis and comparisons undertaken using the log_10_ means (i.e., corresponding to the geometric means of the untransformed data) as measures of central location. For all three NAE lipids, the mean concentration in the high WAZ group was significantly lower compared to the low WAZ group (all *p* < 0.02, critical value of *p* = 0.05). The mean values for the high group was on average 0.19 log_10_ units lower, corresponding to a 35% lower geometric mean of the absolute values in the high versus low WAZ group.

In the two-way ANOVA matching for NAE structure, there was a significant main effect of WAZ group (*p* = 0.002) on the NAE concentration. The dataset failed Mauchly’s test for sphericity; consequently Greenhouse–Geisser corrections were used, resulting in *p* < 0.001 for the main effect of NAE structure, and *p* = 0.380 for the interaction WAZ group × NAE structure. The lack of a significant interaction indicates that there is no evidence for different results for the different NAEs, i.e., the three NAE structures (OEA, SEA, and PEA) did not behave differently from each other.

The ROC curves were constructed for each NAE, and the area under the curve (AUC) was calculated. The data for the three lipids are shown in the lower row graphs in Figure 2. Unsurprisingly, the pattern of significance seen with the parametric *t*-tests was also seen in the ROC curves for all three NAEs.

Spearman’s ρ and the corresponding *p*-values for the 21 correlations obtained for each NAE are shown in Supplementary Figure S1. The three NAEs were highly correlated with one another, but did not correlate with macronutrients in the milk (fat, lactose, protein, total solid matter, or energy; data available for 62 of the 100 samples). In general, measures of infant growth were negatively correlated to the NAE levels, which are further detailed in the following section.

### 3.3. NAEs and Infant Anthropometry and Growth

The mean infant birth weight and the distribution of mBMI were significantly different between the two WAZ groups (Table 1). In order to investigate whether the difference in NAE concentrations between the low and high WAZ group was retained when these covariates were taken into account, multivariate logistic regressions were undertaken. For the whole dataset, the coefficients for log_10_[SEA] and log_10_[PEA] were significant, as were the coefficients for mBMI and birth weight, whereas the coefficients for sex and the season of sampling (October–March vs. April–September) were not (Supplementary Table S1). In the case of OEA, the coefficient for log_10_[OEA] did not reach significance, and a similar *p*-value (0.084) was seen upon exclusion of the presumed outlier. However, the data for OEA is underpowered (explained in the next paragraph).

Supplementation with infant formula within breastfeeding establishment, i.e., early infant formula, also differed between the two groups. In this case, data were available for 81 of the 100 individuals. Given the smaller size of the dataset including this variable, we determined how robust the difference between the log_10_[NAE] concentrations (i.e., OEA, SEA, and PEA individually) in the two WAZ groups was at this sample size. This was undertaken by bootstrapping the data to generate 81 random samples from the 100 sample dataset, and then running a multivariate logistic regression analysis with log_10_[NAE], mBMI, and birth weight as variables to see how often the coefficient for the log_10_[NAE] was significant. Using 1000 iterations and the glm function available in R, *p* < 0.05 was seen in 32.6%, 69.5%, and 55.2% of the cases for OEA, SEA, and PEA, respectively. In consequence, we undertook the logistic regression analysis for the true dataset for 81 individuals to log_10_[SEA] alone, since these data were the most robust with *p* < 0.05 in 69.5% of the cases. The coefficient for log_10_[SEA] remained significant, even when early infant formula was taken into account. The data are shown in Table 2.

Finally, concerning the exclusivity of breastfeeding at the time of sampling, i.e., if the infant was receiving any other food than mother’s milk (e.g., infant formula or complementary foods), we speculated that exclusively breastfed infants were receiving more mother’s milk than their partially breastfed counterparts. Information on breastfeeding exclusivity was available for 82 of the samples in the dataset, and even when including this in the logistic regression analysis, the coefficient for log_10_[SEA] was significant (OR 0.05, 95% CI = 0.00, 0.88, *p* = 0.041).

Due to insufficient amounts of sample material, macronutrient analysis was performed on 62 of the 100 milk samples. However, at this sample size, bootstrapping the data as described above, *p* < 0.05 was found in only 29.1%, 57.3%, and 43.2% of the 1000 iterations for OEA, SEA, and PEA, respectively, indicating that the sample size is underpowered for multivariate logistic regression analysis using macronutrients as covariates; and even with SEA, the analysis will be underpowered. In the actual 62 samples, the log_10_[total NAE] concentrations in the two WAZ groups did not differ significantly (*p* ≥ 0.4).

As the secondary analysis, associations between log_10_[SEA] and other growth-related outcomes were investigated. Given the significant Spearman’s correlation coefficients between the log_10_[SEA] and several of these outcomes (Supplementary Figure S1), significant associations can be expected using a linear regression model. In the unadjusted analysis, log_10_[SEA] as the explanatory variable was inversely associated with several concurrent anthropometric measures; abdominal circumference in cm (β = −3.13, *p* = 0.008), weight in g (β = −1.36, *p* = 0.001), length in cm (β = −2.28, *p* = 0.025), triceps skinfold thickness in mm (β = −1.55, *p* = 0.018), HAZ (β = −1.10, *p* = 0.006), WAZ (β = −1.73, *p* < 0.001), BMIZ (β = −1.51, *p* < 0.001), WHZ (β = −1.40, *p* < 0.001), total weight gain since birth in g (β = −900.66, *p* = 0.011), and weight gain per day in g (β = −6.66, *p* = 0.010), but not to subscapular skinfold thickness in mm (β = −0.52, *p* = 0.294); critical value of *p* = 0.045. The pattern was retained for current weight, WAZ, WHZ, BMIZ, and total weight gain since birth (all *p* < the critical value of *p* = 0.013) in the adjusted model including mBMI, birth weight, and early infant formula as covariates (*n* = 81). Triceps skinfold thickness (*p* = 0.016), subscapular skinfold thickness (*p* = 0.048), weight gain per day (*p* = 0.024), and change in WAZ (Δ WAZ) since birth (*p* = 0.066) were not statistically significant according to the critical value of *p*, see Table 3.

As for the latter, Δ WAZ, we did a post-hoc analysis, where children with a Δ WAZ above the group mean were categorized as high weight gainers. We investigated the proportion of high weight gainers in the low and high WAZ group, respectively. Fourteen (28%) of the low WAZ group were high weight gainers, i.e., had a Δ WAZ above the group mean, and 14 (28%) of the high WAZ group had a Δ WAZ below the group mean. We believe this is partly responsible for the lack of significance regarding the observed associations between SEA and Δ WAZ.

## 4. Discussion

In the present study, we aimed to determine the concentration of NAE lipids—OEA, SEA, and PEA—in human milk samples collected at four months of age, and investigate associations to concurrent infant anthropometry as well as growth from birth. Mothers to infants with lower WAZ had a significant higher concentration of satiety factors OEA, PEA, and SEA in their milk compared to mothers to infants with higher WAZ. We observed significant inverse associations between NAE levels and anthropometric measures (in terms of weight, WAZ, WHZ, and BMIZ) at the time of milk sample collection, as well as growth from birth (in terms of total weight gain since birth), even after adjustment for possible confounders.

At the outset, it is important to consider the main strengths and weaknesses of the study. The strengths of the study are that the samples are from a well characterized cohort, the NAE analysis methodology is well validated, and the data are novel. The main weakness of the study is that the collection of the milk samples, collected as part of the cohort protocol, was not ideal for the present study.

With respect to the latter, most work on NAE and endocannabinoid stability and reproducibility of analysis has been undertaken in plasma samples, where levels of the related NAE (and endocannabinoid) AEA are very sensitive to ex vivo conditions due to release from intact cells in the samples [32]. In our hands, 45 min storage of plasma samples at 4 °C produced the expected increase in AEA levels, but also of OEA levels, whereas SEA levels were not affected [33]. The large variation in NAE concentrations between individuals is also seen in plasma [33,34]. In theory, such a large variation could be due to a measurement artefact. However, for SEA and OEA, measurement of plasma levels in separate batches by the current method gives a very high reproducibility [33], and in the case of AEA, the reproducibility between two different analysis methods is very high [34].

To our knowledge, only one study has investigated the stability of NAEs in human milk. The study group (which included two of us, S.G.-F. and M.D.) investigated NAE and oxylipin levels in three samples from the same mother stored for up to seven days prior to analysis [20]. Consistent with the blood plasma studies, levels of AEA and the related endocannabinoid 2-AG increased rapidly, i.e., a nearly five-fold increase was seen after one day of storage at +4 °C. OEA and PEA levels were not affected at this time point, but increased about five-fold after one week of storage, whilst SEA levels were about 2.5- and 3.5-fold higher after 1 and 7 days of storage, respectively. In contrast, the samples were stable for three months at −80 °C. This was a small study on few milk samples from the same mother, but it raises the caveat that the observed changes in the present study could simply be due to differences in the storage times of the milk samples from the two groups. A simple linear regression of our log_10_[NAE] data vs. days at +4 °C from the mean values published by Wu et al. [19] gave slope values of 0.11, 0.06, and 0.11 log_10_[pmol/L] per day for OEA, SEA, and PEA, respectively. Although extremely approximate, this suggests that the observed log_10_[nmol/L] differences between the low and high WAZ group (0.19, 0.23, and 0.14 for OEA, SEA, and PEA, respectively) observed in the present study would require differences in storage times at +4 °C of ~2, ~3, and ~1 days for OEA, SEA, and PEA, respectively, if explained solely by the sampling conditions. Such large differences in sampling times, particularly for SEA, are highly unlikely. Furthermore, if the abovementioned differences were due to differences in storage time, this would require a systematic error, e.g., where samples from mothers to lower WAZ infants were stored for a longer time than samples from mothers to higher WAZ infants. This is not likely, since the mother-infant dyads attended the physical examination (and thereby the milk sample collection) simply ordered by their cohort identification number.

A second criticism of the present study is that milk sample collection was at a single time-point. However, in a recent study published in this journal, OEA and PEA levels did not significantly differ from two to four weeks postpartum (*n* = 24). In that study, milk sample collection was standardized; two hours of fasting prior to expression, full expression of one breast using an electronic breast pump between 6 a.m. and 10 a.m. Levels of OEA and PEA were of the same order of magnitude as in our study, but levels of SEA were not reported [35]. To our knowledge, no studies have examined the circadian variation of the concentration of NAEs in human milk, which could be another issue [36].

The primary finding in the present study is the demonstration of an inverse association between the NAE levels and the WAZ groups, i.e., higher NAE level is associated to lower WAZ. NAEs are synthesized from *N*-acylphosphatidylethanolamines (NAPEs) through the *N*-acyltransferase/*N*-acylphosphatidylethanolamine-hydrolyzing phospholipase D (NAPE-PLD) pathway [17,37]. The fact that the three NAE levels are highly correlated with each other and that there was no significant interaction between WAZ group and NAE structure in the analyses, suggests that the association may reflect a difference in the catalytic activity of one of the key enzymes responsible for the production of NAEs in milk, although this suggestion requires further investigation in future studies. Nonetheless, the consequence affects the levels and thereby the biological activities of the NAEs, as further examined below.

As stated in the Section 1, OEA—the more studied NAE—leads to prolonged inter-meal intervals, reduced size of feedings, delayed gastric emptying and intestinal transit, increased satiety through an increased fatty acid uptake and a higher oxidation rate. Some controversies exist regarding the intestinal response, since some reports indicate that feeding (especially dietary fat) activates the endogenous formation of OEA in the enterocytes of the small intestine [14], whereas others report a decrease in the small intestinal levels of OEA, PEA, and linoleoylethanolamide (LEA) in a time- and dose-dependent manner by dietary fat [13]. In addition, some indications exist, that CD36 (fatty acid translocase) gene polymorphisms correlate to plasma lipid level variations, i.e., CD36 gene polymorphisms lead to OEA synthesis inability, thereby being more prone to developing metabolic syndrome including obesity [38]. This is supported by the finding that OEA reduces plasma cholesterol and triglyceride levels in rodents [11]. OEA is considered a functional AEA antagonist, suppressing the appetite by stimulating satiety without altering the total motor activity [38], level of anxiety, alertness, or stress [12]. Other mechanisms described are the binding of OEA to glucagon-like peptide 1 (GLP-1), thereby increasing the anorectic properties of GLP-1 [12], but again some discrepancy exists, and others report that satiety signals like GLP-1, ghrelin, cholecystokinin (CKK), and peptide Y (PYY) are—in animal models—not affected by OEA [11].

SEA has been reported to inhibit food intake by downregulating the gene expression of a specific liver enzyme, stearoyl-coenzyme A desaturase-1 (SCD-1) [8]. SCD-1 is involved in the synthesis of monounsaturated fatty acids. SCD-1 deficient mice are lean and hypermetabolic, and leptin-deficient obese mice are significantly less obese when crossed with mice carrying a SCD-1 mutation. It has been suggested that downregulation of SCD-1 is an important component of the anorectic effect of leptin [16]. Orally administered SEA did not result in changes in serum glucose or serum leptin levels, and the degradation products of SEA (ethanolamine and stearic acid) are inactive in reducing food intake, as was also the case with OEA and its’ degradation products [16].

PEA is primarily known for its anti-inflammatory effects [36]. Regarding an anorectic effect, findings are conflicting, but it is probable, that PEA has a less potent effect than OEA [17]. Interestingly, in clinical studies investigating the analgesic effects of PEA, weight loss (as a side effect) was not reported [13].

In our study, the geometric mean OEA and SEA concentrations found in the milk samples were 2.6 nmol/L and 2.1 nmol/L, respectively, in the low; and 1.5 nmol/L and 1.3 nmol/L, respectively, in the high WAZ group. Compared to the concentrations used in the animal experiments (see above), these concentrations are modest. Assuming an exclusively breastfed infant, the average daily milk intake at four months of age would be approx. 100–120 mL/kg, corresponding to a mean intake of 660 mL in the low and 890 mL in the high WAZ group. The total intake of OEA would be ~1.7 nmol in the low and ~1.4 nmol in high WAZ group, respectively, and the total intake of SEA would be ~1.4 nmol in the low and ~1.2 nmol in the high WAZ group, respectively. Since gastrointestinal epithelial cells express both the NAE hydrolytic enzymes NAAH and FAAH, they are well equipped to metabolize NAEs [39,40]. As a consequence, biological effects exerted by NAEs in human milk would presumably have to be mediated directly upon ingestion, rather than following absorption. If it is assumed, for the sake of argument, that the rather low levels in the present study are sufficient to evoke satiety responses in the infants, then these data are consistent with the speculation that infants adjust their intake of milk to the OEA and/or SEA satiety signals.

Finally, it is possible, that the NAEs are not active per se, but simply reflect the pattern of another (unknown) milk component, i.e., being a surrogate marker or variable. Clearly, more work is needed to establish the biological importance of NAEs in human milk.

## 5. Conclusions

In conclusion, the detection of appetite regulators (in this case NAE lipids) in human milk is not a novel finding, but—to our knowledge—this is the largest study investigating the concentration of satiety factors OEA, SEA, and PEA in human milk samples and associations to offspring anthropometry and growth. Based on human milk samples collected at four months of age, we observed statistically significant differences in the concentrations between mothers to infants with a low WAZ and mothers to infants with a high WAZ at time of the milk sample collection. The low WAZ group had a higher concentration of satiety factors OEA, PEA, and SEA compared to the high WAZ group, and even after adjustment for maternal pre-pregnancy BMI, birth weight, and supplementation with infant formula within breastfeeding establishment, a lower concentration of OEA, SEA, and PEA was associated with a higher weight gain since birth. These findings are of great interest in the research area of human milk, appetite regulation, and infant growth, and they could represent another piece of the puzzle of the functions of human milk. In future studies testing the reproducibility of our findings, we emphasize that milk samples should be processed immediately upon collection due to the pronounced ex vivo instability of these compounds.

## Figures and Tables

**Table 1:** Maternal and infant characteristics.

	Lower WAZ	Higher WAZ	*p*
*N*	50	50	
**Infant characteristics**			
Sex ^1^, *n* (%)			
female	23 (46)	22 (44)	>0.99
male	27(54)	28 (56)	
Birth weight, g	3253 ± 544	3894 ± 487	<0.001 *
Birth weight Z-score, SD	−0.7 ± 1.1	0.6 ± 1.1	<0.001 *
Gestational age, days	279 (273–285)	285 (277–290)	0.112 ^2^
Early infant formula ^3^, *n* (%)			
yes	7 (14)	15 (30)	0.048
no	34 (68)	25 (50)	
unknown	9 (18)	10 (20)	
Duration of exclusive breastfeeding, weeks	17.0 (11–19)	19.0 (4–22)	0.105 ^4^
Infant age at time of milk sampling, weeks	17.1 ± 3.0	17.3 ± 3.3	0.735
Infant weight at time of milk sampling, g	6053 (5770–6490)	8140 (7630–8565)	<0.001 *^5^
Exclusive breastfeeding at time of milk sampling, *n* (%)			
yes	17 (34)	15 (30)	0.076
no	24 (48)	26 (52)	
unknown	9 (18)	9 (18)	
Season at milk sampling, *n* (%)			
October–March	19 (38)	21 (42)	0.838
April–September	31 (62)	29 (58)	
**Maternal characteristics**			
Maternal pre-pregnancy BMI (mBMI), kg/m^2^	22.6 (21.1–24.0)	23.5 (21.1–28.7)	0.022 ^6^
Educational level ^7^, *n* (%)			
low	7 (14)	8 (16)	0.519
intermediate	21 (42)	25 (50)	
high	9 (18)	5 (10)	
unknown	13 (26)	12 (24)	
Smoking status, *n* (%)			
no (or stopped during 1st trimester)	50 (100)	49 (98)	n/a
unknown	0 (0)	1 (2)	
Birth type, *n* (%)			
vaginal	41 (82)	38 (76)	0.624
Caesarean section	9 (18)	12 (24)	
Postdelivery parity, *n* (%)			
1	25 (50)	22 (44)	0.457
2	14 (28)	20 (40)	
≥3	11 (22)	8 (16)	

Continuous variables are presented as mean ± SD if normally distributed, otherwise as median (IQR); normality tested by Shapiro–Wilk W test. Statistical test used is two-sided *t*-test (unless otherwise stated) for continuous variables and Fisher’s exact test for categorical variables. The critical value of *p* at a false discovery rate of 5% was 0.008. *p*-values below this critical value are indicated with an asterisk. ^1^ Two-way ANOVA stratifying for WAZ group and sex gave *p* of 0.064, <0.001, and 0.130 for the main effects of sex, WAZ group, and the interaction sex × WAZ group, respectively; ^2^ *p* is for a two-sample Kolmogorov–Smirnov test for difference in distributions, since none of the distributions were normal; Shapiro–Wilk W test *p* < 0.001 and *p* = 0.011 for low and high WAZ, respectively; ^3^ Supplementation with infant formula within breastfeeding establishment, i.e., first few days after birth; ^4^ *p* is for two-sample Kolmogorov–Smirnov test; Shapiro–Wilk W test *p* = 0.004 and *p* < 0.001 for low and high WAZ; ^5^ *p* is for a two-sample Kolmogorov–Smirnov test; Shapiro–Wilk W test *p* = 0.039 and *p* = 0.873 for low and high WAZ; ^6^ *p* is for a two-sample Kolmogorov–Smirnov test; Shapiro–Wilk W test *p* = 0.561 and *p* < 0.001 for low and high WAZ; ^7^ Based on the highest, completed education; low = lower and upper secondary school or vocational education and training; intermediate = short-cycle higher education or medium-cycle higher education; high = long-cycle higher education (i.e., university).

**Table 2:** Multivariate logistic regressions with higher WAZ as outcome.

	Estimate	OR (95% CI)	*p*
**Complete dataset, *n* = 100**			
log_10_[SEA] (log_10_ pmol/L)	−2.82	0.06 (0.01, 0.50)	0.009 *
birth weight (g)	0.00	1.00 (1.00, 1.00)	<0.001 *
mBMI (kg/m^2^)	0.21	1.24 (1.05, 1.45)	0.009 *
**Reduced dataset; as above + information on early infant formula, *n* = 81**			
log_10_[SEA] (log_10_ pmol/L)	−3.49	0.03 (0.00, 0.51)	0.015 *
birth weight (g)	0.00	1.00 (1.00, 1.00)	0.002 *
mBMI (kg/m^2^)	0.20	1.22 (1.01, 1.47)	0.039 *
early infant formula ^1^	0.88	2.42 (0.52, 11.30)	0.261

^1^ Supplementation with infant formula within breastfeeding establishment, i.e., first few days after birth. The critical value of *p* at a false discovery rate of 5% was 0.043. *p*-values below this critical value are indicated with an asterisk.

**Table 3:** Multivariate linear regressions for anthropometric and growth outcome measures.

Explanatory Variables	log_10_[SEA] (log_10_ pmol/L)		mBMI	Birth Weight	Early IF ^1^
Outcome Measure	β (95% CI)	*p*	*p*	*p*	*p*
Abdominal circumference (cm)	−2.28 (−5.28, 0.71)	0.134	0.511	<0.001 *	0.288
Weight at sampling (g)	−1.38 (−2.35, −0.41)	0.006 *	0.098	<0.001 *	0.201
Length at sampling (cm)	−1.28 (−3.68, 1.11)	0.290	0.955	<0.001 *	0.938
Triceps skinfold thickness (mm)	−2.24 (−4.04, −0.43)	0.016	0.089	0.435	0.926
Subscapular skinfold thickness (mm)	−1.27 (−2.53, −0.01)	0.048	0.009 *	0.543	0.259
WAZ (SD)	−1.56 (−2.56, −0.56)	0.003 *	0.110	<0.001 *	0.203
HAZ (SD)	−0.37 (−1.25, 0.52)	0.411	0.846	<0.001 *	0.784
WHZ (SD)	−1.85 (−2.85, −0.85)	<0.001 *	0.021	0.174	0.086
BMIZ (SD)	−1.83 (−2.85, −0.81)	<0.001 *	0.030	0.037	0.142
∆ weight since birth (g)	−1381 (−2,350, −413)	0.006 *	0.098	0.698	0.201
∆ weight since birth per day (g)	−8.17 (−15.26, −1.08)	0.024	0.183	0.587	0.142
Δ WAZ since birth (SD)	−1.04 (−2.15, 0.07)	0.066	0.167	0.001 *	0.320

^1^ IF = infant formula; supplementation with infant formula within breastfeeding establishment, i.e., first few days after birth. Data shown are for the fully adjusted model including log10[SEA], mBMI, birth weight, and early infant formula as explanatory variables (*n* = 81). Residual plots were acceptable in all cases. For the covariates included, only *p* is shown (three last columns). The critical value of *p* at a false discovery rate of 5% was 0.013. *p*-values below this critical value are indicated with an asterisk.

## Supplementary Materials

The following are available online at http://www.mdpi.com/2072-6643/10/11/1747/s1, Figure S1: Correlations between OEA, SEA, and PEA and maternal and infant characteristics; Table S1: Logistic regressions for log_10_[NAE] with higher WAZ as outcome.
