# Supplementary material for: Satiety Factors Oleoylethanolamide, Stearoylethanolamide, and Palmitoylethanolamide in Mother’s Milk Are Strongly Associated with Infant Weight at Four Months of Age—Data from the Odense Child Cohort
Source: Nutrients. 2018 Nov 13;10(11):1747. doi: 10.3390/nu10111747 (PMC6266120; doi:10.3390/nu10111747)
Supplement: Supplementary file 1 [file nutrients-10-01747-s001.pdf]

Supplementary Table S1 – Logistic regressions for $\log_{10}$[NAE] with higher WAZ as outcome

	estimate	OR (95% CI)	*P*
OEA			
$\log_{10}$[OEA] ($\log_{10}$ pmol/L)	-1.07	0.34 (0.10, 1.16)	0.084
birth weight (g)	0.00	1.00 (1.00, 1.00)	<0.001
mBMI (kg/m^2)	0.20	1.22 (1.04, 1.43)	0.015
season	-0.38	0.68 (0.23, 2.00)	0.487
sex	-0.37	0.69 (0.25, 1.95)	0.488
SEA			
$\log_{10}$[SEA] ($\log_{10}$ pmol/L)	-2.81	0.06 (0.01, 0.52)	0.010
birth weight (g)	0.00	1.00 (1.00, 1.00)	<0.001
mBMI (kg/m^2)	0.21	1.23 (1.05, 1.44)	0.012
season	-0.34	0.71 (0.24, 2.17)	0.553
sex	-0.13	0.88 (0.30, 2.60)	0.820
PEA			
$\log_{10}$[PEA] ($\log_{10}$ pmol/L)	-2.52	0.08 (0.01, 0.74)	0.026
birth weight (g)	0.00	1.00 (1.00, 1.00)	<0.001
mBMI (kg/m^2)	0.20	1.23 (1.05, 1.44)	0.012
season	-0.31	0.73 (0.25, 2.18)	0.578
sex	-0.21	0.81 (0.28, 2.31)	0.691

Data shown are for the fully adjusted model including all explanatory variables mentioned (n = 100). For season, summer is the reference; for sex, female is the reference.
The critical value of *P* at a 5% false discovery rate was 0.027.

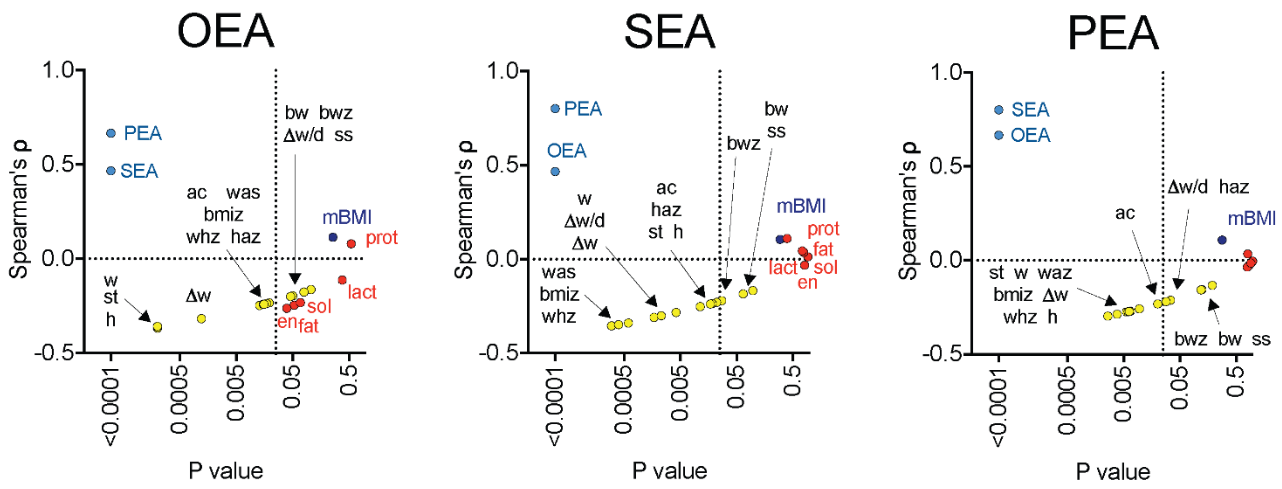

Figure S1: Correlations between OEA, SEA, and PEA and maternal and infant characteristics
